# Prostaglandin E_2_/Leukotriene B_4_ balance induced by *Lutzomyia longipalpis* saliva favors *Leishmania infantum* infection

**DOI:** 10.1186/s13071-014-0601-8

**Published:** 2014-12-20

**Authors:** Théo Araújo-Santos, Deboraci Brito Prates, Jaqueline França-Costa, Nívea F Luz, Bruno B Andrade, José Carlos Miranda, Claudia I Brodskyn, Aldina Barral, Patrícia T Bozza, Valéria Matos Borges

**Affiliations:** Gonçalo Moniz Research Center, Oswaldo Cruz Foundation (FIOCRUZ), Salvador, BA Brazil; Present address: Center of Biological Sciences and Health, Federal University of Western Bahia, Barreiras, BA Brazil; Federal University of Bahia (UFBA), Salvador, BA Brazil; Departamento de Biomorfologia, Instituto de Ciências da Saúde, Universidade Federal da Bahia, 40110-100 Salvador, BA Brazil; Immunobiology Section, Laboratory of Parasitic Diseases, National Institute of Allergy and Infectious Diseases, National Institutes of Health, 20893 Bethesda, MD USA; Institute for Investigation in Immunology, iii-INCT (National Institute of Science and Technology), São Paulo, Brazil; Oswaldo Cruz Institute, Oswaldo Cruz Foundation, Rio de Janeiro, RJ Brazil

**Keywords:** *Lutzomyia longipalpis*, *Leishmania infantum*, Saliva, Prostaglandina E_2_, Leukotriene B_4_

## Abstract

**Background:**

Eicosanoids and sand fly saliva have a critical role in the *Leishmania* infection. Here, we evaluated the effect of *Lutzomyia longipalpis* salivary gland sonicate (SGS) on neutrophil and monocyte recruitment and activation of eicosanoid production in a murine model of inflammation.

**Methods:**

C57BL/6 mice were inoculated intraperitonealy with *Lutzomyia longipalpis* SGS or *Leishmania infantum* or both, followed by analyses of cell recruitment, parasite load and eicosanoid production.

**Results:**

Intraperitoneal injection of *Lutzomyia longipalpis* SGS together with *Leishmania infantum* induced an early increased parasite viability in monocytes and neutrophils. *L. longipalpis* SGS increased prostaglandin E_2_ (PGE_2_), but reduced leukotriene B_4_ (LTB_4_) production *ex vivo* in peritoneal leukocytes. In addition, the pharmacological inhibition of cyclooxygenase 2 (COX-2) with NS-398 decreased parasite viability inside macrophages during *Leishmania* infection in the presence of *L. longipalpis* SGS arguing that PGE_2_ production is associated with diminished parasite killing.

**Conclusions:**

These findings indicate that *L. longipalpis* SGS is a critical factor driving immune evasion of *Leishmania* through modulation of PGE_2_/LTB_4_ axis, which may represent an important mechanism on establishment of the infection.

## Background

*Leishmania infantum* in America is transmitted by the bite of infected *Lutzomyia longipalpis* sand flies. Transmission of *Leishmania* sp. by hematophagous sand fly vectors occurs during blood feeding, when salivary content is inoculated with regurgitated *Leishmania* into host skin. Sand fly saliva enhances *Leishmania* infection on several experimental models [1-3] through its modulatory effects on the host immune system [4,5]. A successful blood feeding depends on the formation of a blood hemorrhagic pool [6]. In such a microenvironment there are many inflammatory cells [4], and *L. longipalpis* saliva has been shown to enhance recruitment of different cells, including monocytes and neutrophils [7-10].

Eicosanoids display an important role during *Leishmania* infection [11-16]. In this context, there are results showing that Prostaglandin E_2_ (PGE_2_) production benefits parasite survival [15-18] while Leukotriene B_4_ (LTB_4_) is related with parasite killing by host cells [12,14,19]. In addition, sand fly saliva seems to modulate the eicosanoid production by host cells in a polarized way towards PGE_2_ [10,11,15,20]. Maxadilan, a vasodilatory peptide present in *L. longipalpis* salivary glands, is shown to increase production of PGE_2_ by macrophages [21]. *L. longipalpis* salivary gland sonicate (SGS) is able to modulate PGE_2_ and LTB_4_ release in monocytes and neutrophils recruited to the peritoneal cavity [20]. In neutrophils, SGS increases *L. infantum* infection-driven production of PGE_2_*in vitro* [15]. However, it remains to be addressed whether sand fly saliva can benefit *Leishmania* infection by control of PGE_2_/LTB_4_ axis during early steps of infection *in vivo*.

In the present study, we explore the effect of *L. longipalpis* SGS on the PGE2/LTB4 balance in the context of *L. infantum* infection *in vivo* using the peritoneal model in mice. In addition, we demonstrate that PGE2/LTB4 balance can be important for modulation of immune response elicited by SGS allowing increase in parasite viability as well as parasite burden inside leukocytes during early moments of exposure to *L. infantum*.

## Methods

### Antibodies and reagents

Schneider’s insect medium, N-(1-naphthyl)-ethylenediamine and p-Aminobenzene-sulfanilamide were purchased from SIGMA (St. Louis, MO). RPMI 1640 medium and L-glutamine, penicillin, and streptomycin were from Invitrogen (Carlsbad, CA, USA). Nutridoma-SP was from Roche (Indianapolis, In, USA). A23187 calcium ionophore was from Calbiochem Novabiochem Corp. (La Jolla, CA). NS-398, PGE_2_ and LTB_4_ enzyme-linked immunoassay (EIA) Kits were from Cayman Chemical (Ann Arbor, MI). Dimethylsulfoxide (DMSO) was purchased from ACROS Organics (New Jersey, NJ).

### Ethics statement

All experiments were performed in strict accordance with the recommendations of the Brazilian National Council for the Control of Animal Experimentation (CONCEA). The Ethics Committee on the use of experimental animals (CEUA) of the Centro de Pesquisas Gonçalo Moniz, Fundação Oswaldo Cruz approved all protocols (Permit Number: 27/2008).

### Animals

Inbred male C57BL/6 mice, age 6–8 weeks, were obtained from the animal facility at Centro de Pesquisas Gonçalo Moniz, Fundação Oswaldo Cruz (CPqGM-FIOCRUZ, Bahia, Brazil). The animals were kept at a temperature of 24°C, with free access to food and water and light and dark cycles of 12 hours each.

### Parasite

*L. infantum* (MCAN/BR/89/BA262) promastigotes were cultured at 25°C in Schneider’s insect medium supplemented with 20% inactive FBS, 2 mM L-glutamine, 100 U/ml penicillin, and 100 μg/ml streptomycin. Stationary phase promastigotes were used in all experiments.

### Sand flies and preparation of salivary glands

Adult *Lutzomyia longipalpis* captured in Cavunge (Bahia, Brazil) were reared at the Laboratório de Imunoparasitologia/CPqGM/FIOCRUZ (Bahia, Brazil) as described previously [8]. Salivary glands were dissected from 5- to 7-day-old *L. longipalpis* females under a stereoscopic microscope (Stemi 2000, Carls Zeiss, Jena, Germany) and stored in groups of 10 pairs in 10 μl endotoxin-free PBS at −70°C. Immediately before use, glands were sonicated (Sonifier 450, Brason, Danbury, CT) and centrifuged at 10,000 × g for 4 minutes. The supernatants of salivary gland sonicate (SGS) were used for the experiments. The level of LPS contamination of SGS preparations was determined using a commercially available LAL Chromogenic Kit (QCL-1000, Lonza Bioscience) resulting in negligible levels of endotoxin in the salivary gland supernatant. All experimental procedures used SGS equivalent to 0.5 pair of salivary gland per group which possesses approximately 0.7 micrograms of proteins [22].

### Mice infection

C57BL/6 mice were submitted to intra-peritoneal (i.p.) injection with 0.1 ml of SGS (0.5 pair/cavity), 0.1 ml of *L. infantum* promastigotes from stationary phase (3 × 10^6^/cavity), 0.1 ml of endotoxin-free saline per cavity (negative control) or 0.1 ml of LPS (20 μg/ml; positive control-data not shown). One hour after the stimulus the total leukocytes that migrated to the peritoneal cavity was harvested by peritoneal lavage with injection of 10 ml endotoxin-free saline. Alternatively, C57BL/6 mice were previously treated with an i.p. injection of NS398 2 mg/kg or DMSO as a vehicle control. Total counts were performed on a Neubauer hemocytometer after staining with Turk’s solution. Differential cell counts (200 cells total) of infected cells were carried out microscopically on cytospin preparations stained with Diff-Quick.

### Assessment of intracellular load of *L. infantum*

Intracellular load of *L. infantum* was estimated by production of proliferating extracellular motile promastigotes in Schneider medium [23]. Briefly, after 1 h of infection, peritoneal cells were centrifuged, supernatants containing non-internalized promastigotes were removed and medium was replaced by 250 μl of Schneider medium supplemented with 20% inactive FBS, 2 mM L-glutamine, 100 U/ml penicillin, and 100 μg/ml streptomycin. Infected cells were cultured at 25°C for additional 3 days. In the third day promastigotes in the cultures were counted in a Neubauer hemocytometer.

### Transmission electron microscopy

Peritoneal cells from mice infected with *L. infantum* were centrifuged (1500 rpm, 10 min) and the pellets were resuspended and fixed in a mixture of freshly prepared aldehydes (1% paraformaldehyde and 1% glutaraldehyde) in 0.1 M phosphate buffer, pH7.4 overnight at 4°C. The cells were washed in the same buffer and embedded in molten 2% agar (Merk). Agar pellets containing the cells were post-fixed in a mixture of 1% phosphate-buffered osmium tetroxide and 1.5% potassium ferrocyanide (final concentration) for 1 h and processed for resin embedding (PolyBed 812, Polysciences, Warrington, PA). The section were mounted on uncoated 200-mesh copper grids and observed through a transmission electron microscope (EM 109; Zeiss, Germany). Electron micrographs were randomly taken at the magnifications of 7 000 – 30 000X to study the entire cell profile. Viable parasites inside infected neutrophils and monocytes were enumerated by electron microscopy and presented as parasite viability index which is the ratio of percentage of viable parasite by the mean of parasites per infected cells. Parasites were considered dead according chromatin condensation and presence of cytoplasmic disorganization and vacuolization.

### PGE_2_ and LTB_4_ measurements

PGE_2_ and LTB_4_ levels were measured *ex vivo* from leukocytes harvested by peritoneal cavity washing with Ca^2+^-Mg^2+^-free HBSS. Afterwards, recovered cells (1 × 10^6^ cells/ml) were resuspended in HBSS contained Ca^2+^-Mg^2+^ and then stimulated with A23187 (0.5 μM) for 15 min. Reactions were stopped on ice, and samples were centrifuged at 500 × g for 10 min at 4°C. Supernatants were collected to measure PGE_2_ and LTB_4_ by enzyme-linked immunoassay (EIA), according to manufacturer’s instructions.

### Statistical analysis

The assays were performed using at least five mice per group. Each experiment was repeated at least three times. The data are presented as the mean and SEM (standard error) of representative experiments and were analyzed using the GraphPad Prism 5.0 software (GraphPad Software, San Diego, CA, USA). Kinetic data were alternatively shown as bars graphs with area under curves calculated using GraphPad Prism 5.0 software. The comparisons between two groups were analyzed using Mann–Whitney test. The differences were considered statistically significant when p ≤ 0.05.

## Results

*L. longipalpis* SGS and *L. infantum* modulate early leucocyte recruitment and eicosanoids production *ex vivo.*

**Figure 1:**
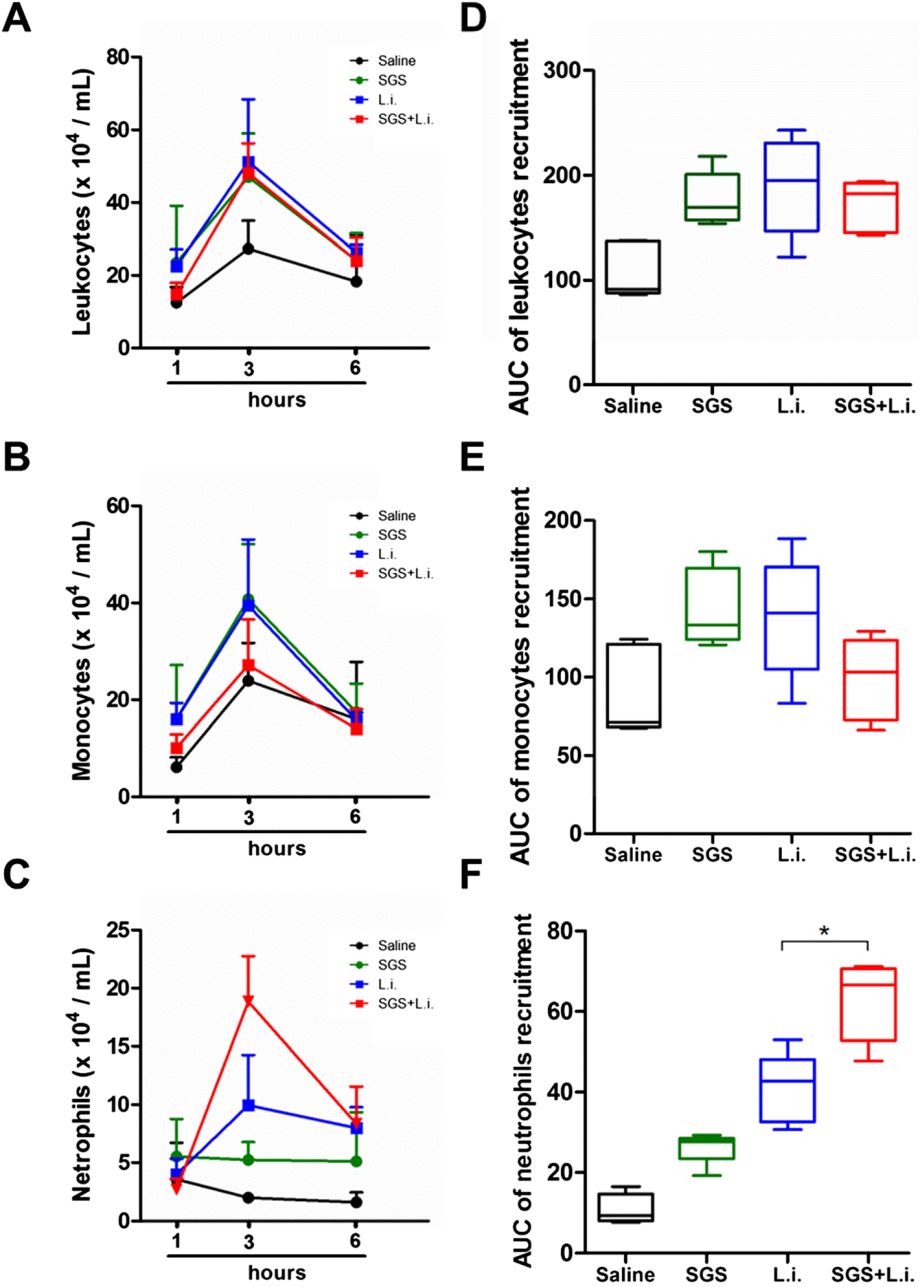
**Leukocyte recruitment in response to** ***L. longipalpis*** **SGS during** ***L. infantum*** **infection.** C57BL/6 mice were injected i.p. with saline (control), *L. infantum* and/or SGS as described in methods. One, 3 and 6 hours after stimulation, peritoneal cavities were washed and cells were harvested. Kinetics of total leucocytes **(A)**, monocytes **(B)** and neutrophils **(C)** were estimated using Diff-Quick-stained cytospin preparations. Area Under Curve (AUC) of the kinetic of total leukocytes **(D)**, monocytes **(E)** and neutrophils **(F)** recruitment. The data are the means and SEM from an experiment representative of three independent experiments. n = 5 per group. (*, p < 0.05 and Mann–Whitney test, two-tailed).

We have previously shown that the main cell types recruited to the inoculation site by *L. longipalpis* saliva are neutrophils and monocytes [7,8,20]. Here we observed an early recruitment kinetic of total leukocytes (Figure 1A), monocytes (Figure 1B) and neutrophils (Figure 1C) in the peritoneal cavity. *L. infantum* inoculation with SGS did not alter the number of total leukocytes (Figure 1D) and monocytes (Figure 1E), but there was a significant increase in the number of neutrophils (Figure 1F).

Different classes of eicosanoids, such as PGE_2_ and LTB_4_, promote both cellular recruitment and removal of inflammatory cells coordinating the initial events of inflammation [24].

We have previously demonstrated that injection of *L. longipalpis* saliva in the peritoneal cavity induced PGE_2_ and LTB_4_ production [20]. In the present study we investigated whether the presence of *L. infantum* alters the levels of PGE_2_ and LTB_4_ production induced by *L. longipalpis* saliva.

**Figure 2:**
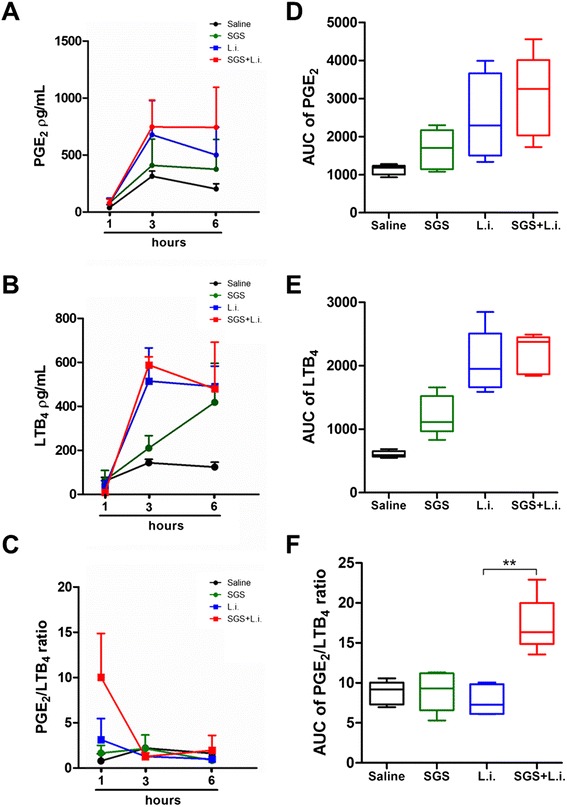
**PGE**_**2**_ **and LTB**_**4**_ **production in response to** ***L. longipalpis*** **SGS during** ***L. infantum*** **infection.** C57BL/6 mice were injected i.p. with saline (control), *L. infantum* and/or SGS. One, 3 and 6 hours after stimulation, peritoneal cavities were washed and cells were harvested. The cells were then incubated with A23187 (0.5 mM) for 15 min at 37°C to evaluate LTB_4_ and PGE_2_ production. The concentrations of PGE_2_ **(A)** and LTB_4_ **(B)** in the supernatant were measured by ELISA. **(C)** Shows the PGE_2_/LTB_4_ ratio. Area Under Curve (AUC) of the kinetic of PGE_2_ **(D)**, LTB_4_ **(E)** and PGE_2_/LTB_4_ ratio **(F)**. The data are the means and SEM from an experiment representative of three independent experiments. n = 5 per group. (*, p < 0.05 and Mann–Whitney test, two-tailed).

Addition of *L. longipalpis* SGS to *L. infantum* inoculation did not alter PGE_2_ (Figure 2A,D) and LTB_4_ (Figure 2B,E) levels significantly at the time points evaluated. Considering the fact that PGE_2_ and LTB_4_ exhibit antagonistic effects on inflammation and *Leishmania* infection [11,12,14-16,25], we plotted the PGE_2_/LTB_4_ ratio, following analysis of Area Under Curve (AUC) of the kinetic of these inflammatory mediators (Figure 2C,F). Injection of *L. longipalpis* salivary components plus *L. infantum* triggered high PGE_2_/LTB_4_ ratio (Figure 2F). However, this additive effect was evident only 1 hour after stimulation (Figure 2C). PGE_2_ levels were undetectable when directly measured in the exudates (not shown).

*L. longipalpis* SGS enhances parasite viability during *L. infantum* infection *in vivo.*

**Figure 3:**
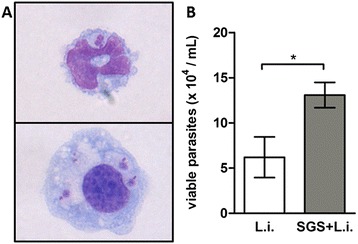
***L. longipalpis*** **SGS favors** ***L. infantum*** **survival inside neutrophils and monocytes.** C57BL/6 mice were inoculated with *L. infantum* and/or SGS. **(A)** Illustration of peritoneal infected neutrophil (upper) and macrophage (bottom) stained with Diff-Quick after 1 h of inoculation. **(B)** Viable parasites counting recovered by total infected peritoneal cells. The data are the means and SEM from an experiment representative of three independent experiments. n = 5 per group. (*, p < 0.05 and Mann–Whitney test, two-tailed).

Infected neutrophils and monocytes were observed in recovered cells from peritoneal cavity after 1 hour of inoculation of *L. longipalpis* SGS plus *L. infantum* (Figure 3A). The number of viable parasites from these cell cultures was increased significantly in the presence of *L. longipalpis* SGS (Figure 3B). Infected neutrophils and monocytes were not observed in later time points than 1 hour in recovered cells from the peritoneal cavity (not shown).

**Figure 4:**
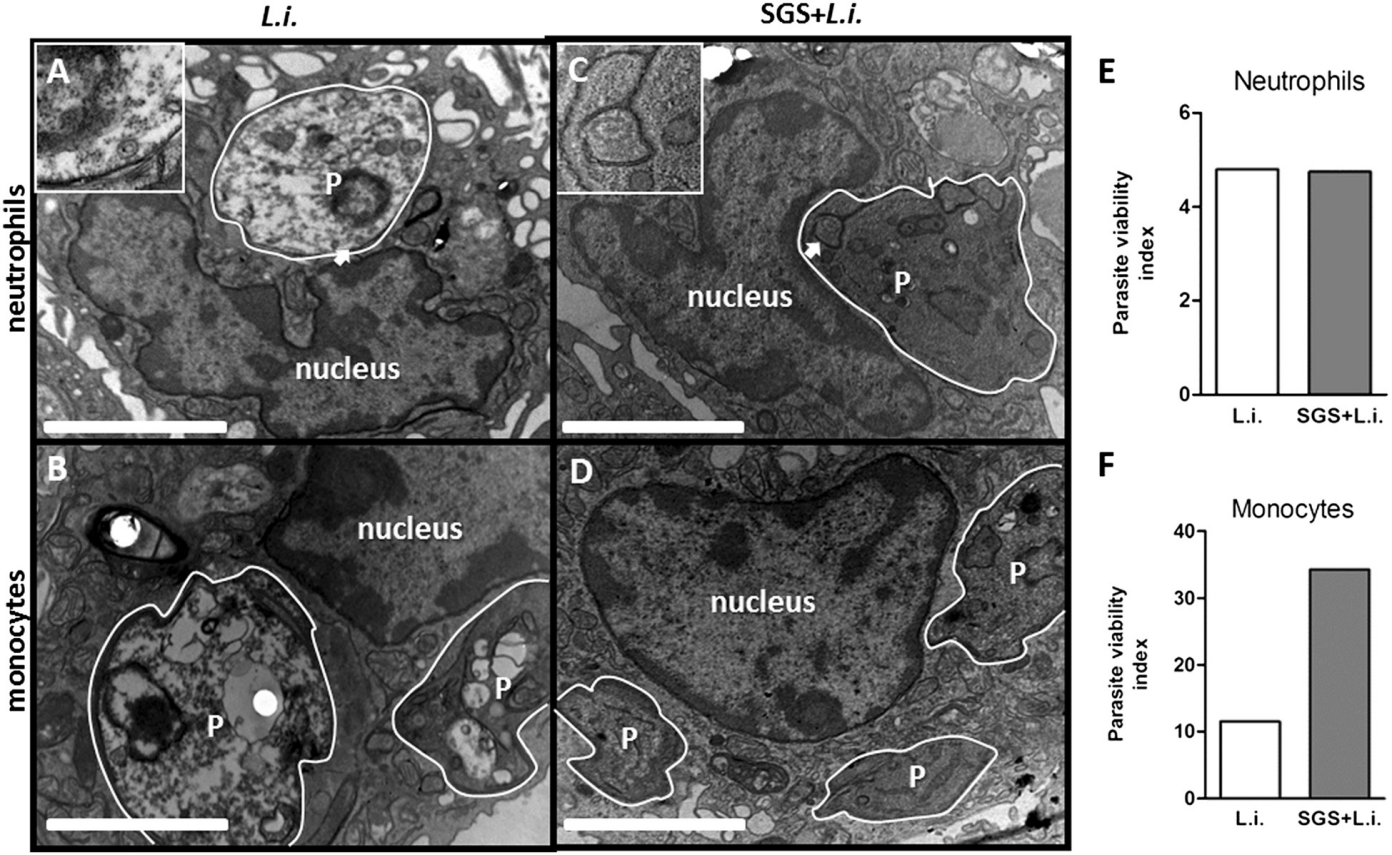
***L. longipalpis*** **SGS favors viability of** ***L. infantum*** **inside peritoneal cells.** C57BL/6 mice were inoculated with *L infantum* and/or SGS. Transmission electron microscopic images of peritoneal cells after 1 h of infection with *L. infantum* are shown. Degraded *L. infantum* inside neutrophils **(A)** and monocytes **(B)** are showed. Viable parasites were observed in neutrophils **(C)** and monocytes **(D)** in those animals infected in the presence of *L. longipalpis* SGS. Insets indicated by white arrowheads shows details of parasite inside parasitophorous vacuoles (PV) outlined in white (50X-fold increase). P- parasite. Viable parasites inside infected neutrophils **(E)** and monocytes **(F)** were enumerated by electron microscopy and presented as parasite viability index calculated as described in Methods section. (*, p < 0.05 and Mann–Whitney test, two-tailed).

We have also evaluated the presence of parasites inside peritoneal neutrophils and monocytes by transmission electron microscopy (Figure 4A-D) after 1 hour post *in vivo* inoculation. Cells recovered from mice injected with *L. infantum* alone frequently presented degenerated parasite with chromatin condensation, cytoplasmic disorganization and vacuolization inside the vacuoles in neutrophils (Figure 4A) and monocytes (Figure 4B). In contrast, when *L. longipalpis* SGS was inoculated together with *Leishmania*, viable parasites were recurrently observed in both neutrophils (Figure 4C) and monocytes (Figure 4D). However, the parasite viability was increased only inside monocytes (Figure 4F), but not neutrophils (Figure 4E) as shown by analysis of electron microscopy sections (Figure 4).

Effect of *L. longipalpis* saliva on PGE_2_ production and *L. infantum* infection *ex vivo.*

**Figure 5:**
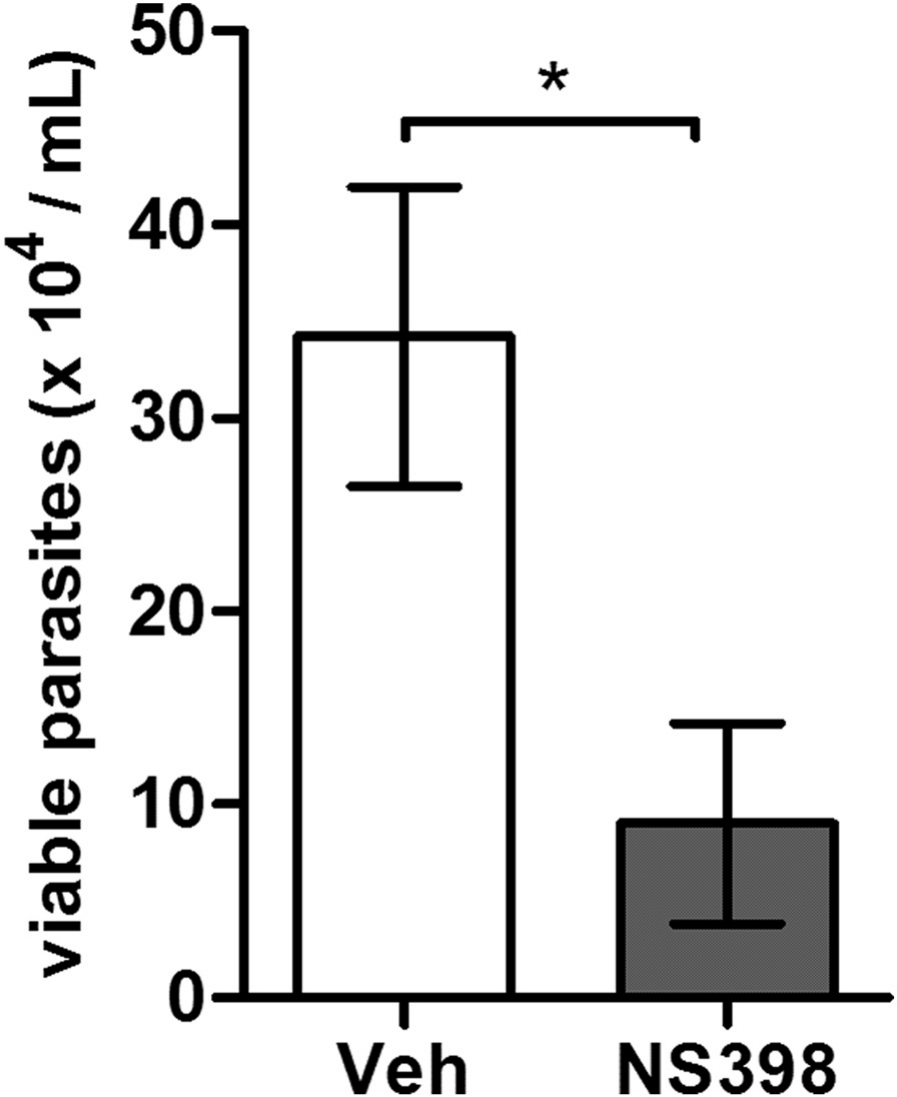
**Ciclooxiganese-2 inhibition affects the parasite viability** ***in vivo*** **during** ***L. infantum*** **infection in the presence of SGS.** C57BL/6 mice were treated with DMSO (vehicle – Veh) or NS398 2 mg/kg. After 1 h of treatment, mice were injected i.p. with *L. infantum* and SGS according to methods. Viable parasites counting recovered by total infected peritoneal cells after 1 h *L. infantum* or *L. infantum* plus SGS inoculation. Graph shows viable parasites counting recovered by total infected peritoneal cells. The data are the means and SEM from one experiment representative of three independent experiments. n = 5 per group. (*, p < 0.05 and Mann–Whitney test, two-tailed).

To assess if the manipulation of the eicosanoid balance driven by SGS is important to *L. infantum* infection *in vivo*, we inhibited pharmacologically the PGE_2_ production by using a cyclooxygenase-2 (COX-2) selective inhibitor NS398. The inhibition of COX-2 decreased the number of viable parasites in peritoneal cells after *L. infantum* infection in the presence of SGS (Figure 5). Taken together, these findings suggest that the balance between PGE_2_ and LTB_4_ levels induced by *L. longipalpis* SGS is associated with increased *L. infantum* infection.

## Discussion

Sand fly saliva displays an important role in the first steps of *Leishmania* infection, as it induces cellular recruitment to inflammatory site, inhibits proinflammatory cytokines and deactivates dendritic cells to mobilize regulatory T cells [5]. Previous studies have shown the participation of eicosanoid in the inflammatory response triggered by sand fly saliva [9,20,21]. Here, we show for the first time that saliva can modulate eicosanoid profile with a balance skewed towards COX-2 driven PGE_2_ over LTB_4_ at early time points post *L. infantum* inoculation, possibly benefiting infection.

PGE_2_ production favors the establishment of several pathogen infections [26]. PGE_2_ drives *L. donovani* infection in macrophages via PGE_2_ receptor 2 (EP2) activation [16]. In rats and mice, *Trypanosoma cruzi* infection induces PGE_2_ by macrophages [27-29]. During *Mycobacterium bovis* infection, the increase of PGE_2_ and TGF-β1 production by macrophages that phagocyte apoptotic neutrophils in the inflammatory site helps the establishment of the infection by this pathogen [30]. In addition, the interaction between human apoptotic neutrophils and macrophages also increases the number of parasite burden in *L. amazonensis* infection via PGE_2_ and TGF-β1 production [31]. On the other hand, LTB4 has opposite effects on the inflammatory response. The production of LTB_4_ is associated to the increase of pathogen killing [32,33]. In the context of *Leishmania* infection, LTB_4_ is involved in nitric oxide production and consequently reduction of parasite burden in different cellular models of infection with *L. amazonensis* [12,14,19,25]. We have previously shown that *L. longipalpis* saliva promptly activates macrophages to produce PGE_2_ but not LTB_4_*in vitro* and *ex vivo* [20]. SGS increases PGE_2_ production by neutrophils during *L. infantum* infection [15]. Here we demonstrate that *L. longipalpis* SGS reduce the early LTB_4_ production during *L. infantum* infection whereas it orchestrates an anti-inflammatory response by increasing PGE_2_ production. Accordingly, in a hamster model of human VL, a role for early PGE_2_ in *L. donovani* dissemination and impairment of NOS2 was demonstrated [34,35]. The inoculation of *L. longipalpis* SGS plus *L. infantum* increased parasite viability inside peritoneal cells. The pharmacological inhibition of COX-2 reversed the effect of SGS on enhancing parasite viability. COX-2 expression can be stimulated early dependent upon stimulus, but we do not discharge the action of NS-398 on COX-1 activity. These data strongly suggest that the presence of sand fly SGS favors an anti-inflammatory balance in the PGE_2_/LTB_4_ axis which could facilitate the parasite transmissibility and infection *in vivo*. Despite the lack of data about eicosanoids during later stages of *L. infantum* infection, recent publication of our group shows increased plasma levels of PGE_2_ in patients with localized and diffuse cutaneous leishmaniasis when compared to healthy individuals [36]. Moreover the lesions of these patients present high levels of COX-2 enzyme and other enzymes associated to prostaglandin production [36], suggesting that eicosanoids balance can be important during different stages of the disease.

## Conclusion

In summary, our findings demonstrate that the eicosanoid profile induced by sand fly saliva plays an important role in the inflammatory modulation during early stages of *L. infantum* infection and point out potential implications of the PGE2/LTB4 balance in the immunopathogenesis of visceral leishmaniasis.

## Abbreviations

SGS: Salivary gland sonicate
L. longipalpis: Lutzomyia longipalpis
L. infantum: Leishmania infantum
PGE_2_: Prostaglandin E_2_
LTB_4_: leukotriene B_4_
COX-2: Cyclooxygenase 2

## References

[1] Samuelson J, Lerner E, Tesh R, Titus R (1991). A mouse model of *Leishmania braziliensis* braziliensis infection produced by coinjection with sand fly saliva. J Exp Med.

[2] Belkaid Y, Kamhawi S, Modi G, Valenzuela J, Noben-Trauth N, Rowton E, Ribeiro J, Sacks DL (1998). Development of a natural model of cutaneous leishmaniasis: powerful effects of vector saliva and saliva preexposure on the long-term outcome of *Leishmania major* infection in the mouse ear dermis. J Exp Med.

[3] Kamhawi S, Belkaid Y, Modi G, Rowton E, Sacks D (2000). Protection against cutaneous leishmaniasis resulting from bites of uninfected sand flies. Science (80- ).

[4] Andrade BB, Teixeira CR, Barral A, Barral-Netto M (2005). Haematophagous arthropod saliva and host defense system: a tale of tear and blood. An Acad Bras Cienc.

[5] Andrade BB, de Oliveira CI, Brodskyn CI, Barral A, Barral-Netto M (2007). Role of sand fly saliva in human and experimental leishmaniasis: current insights. Scand J Immunol.

[6] Ribeiro JM (1987). Role of saliva in blood-feeding by arthropods. Annu Rev Entomol.

[7] Teixeira CR, Teixeira MJ, Gomes RB, Santos CS, Andrade BB, Raffaele-Netto I, Silva JS, Guglielmotti A, Miranda JC, Barral A, Brodskyn C, Barral-Netto M (2005). Saliva from *Lutzomyia longipalpis* induces CC chemokine ligand 2/monocyte chemoattractant protein-1 expression and macrophage recruitment. J Immunol.

[8] Silva F, Gomes R, Prates D, Miranda JC, Andrade B, Barral-Netto M, Barral A (2005). Inflammatory cell infiltration and high antibody production in BALB/c mice caused by natural exposure to *Lutzomyia longipalpis* bites. Am J Trop Med Hyg.

[9] Monteiro MC, Lima HC, Souza AAA, Titus RG, Romão PRT, Cunha FDQ (2007). Effect of *Lutzomyia longipalpis* salivary gland extracts on leukocyte migration induced by *Leishmania major*. Am J Trop Med Hyg.

[10] Carregaro V, Valenzuela JG, Cunha TM, Verri WA, Grespan R, Matsumura G, Ribeiro JMC, Elnaiem DE, Silva JS, Cunha FQ, Verri WA, Jr WAV, Ribeiro MC, Silva S (2008). Phlebotomine salivas inhibit immune inflammation-induced neutrophil migration via an autocrine DC-derived PGE2/IL-10 sequential pathway. J Leukoc Biol.

[11] Prates DB, Araújo-Santos T, Brodskyn C, Barral-Netto M, Barral A, Borges VM (2012). New Insights on the Inflammatory Role of *Lutzomyia longipalpis* Saliva in Leishmaniasis. J Parasitol Res.

[12] Tavares NM, Araújo-Santos T, Afonso L, Nogueira PM, Lopes UG, Soares RP, Bozza PT, Bandeira-Melo C, Borges VM, Brodskyn C (2014). Understanding the mechanisms controlling ***Leishmania amazonensis*** infection in vitro: the role of LTB4 derived from human neutrophils.. J Infect Dis.

[13] Araújo-Santos T, Rodríguez NE, Moura Pontes S, Dixt UG, Abánades DR, Bozza PT, Wilson ME, Borges VM: **Role of prostaglandin F2α production in lipid bodies from*****Leishmania infantum chagasi*****: insights on virulence.** *J Infect Dis* 2014, **210**(12):1951–1961. 10.1093/infdis/jiu299 PMC6281352 24850789

[14] Serezani CH, Perrela JH, Russo M, Peters-Golden M, Jancar S (2006). Leukotrienes are essential for the control of *Leishmania amazonensis* infection and contribute to strain variation in susceptibility. J Immunol.

[15] Prates DB, Araújo-Santos T, Luz NF, Andrade BB, França-Costa J, Afonso L, Clarêncio J, Miranda JC, Bozza PT, Dosreis GA, Brodskyn C, Barral-Netto M, Borges VM, Barral A (2011). *Lutzomyia longipalpis* saliva drives apoptosis and enhances parasite burden in neutrophils. J Leukoc Biol.

[16] Saha A, Biswas A, Srivastav S, Mukherjee M, Das PK, Ukil A (2014). Prostaglandin E2 negatively regulates the production of inflammatory cytokines/Chemokines and IL-17 in visceral leishmaniasis. J Immunol.

[17] Lonardoni MV, Barbieri CL, Russo M, Jancar S (1994). Modulation of *Leishmania (L.) amazonensis* growth in cultured mouse macrophages by prostaglandins and platelet activating factor. Mediat Inflamm.

[18] Pinheiro RO, Nunes MP, Pinheiro CS, D’Avila H, Bozza PT, Takiya CM, Corte-Real S, Freire-de-Lima CG, DosReis GA, Côrte-Real S (2008). Induction of autophagy correlates with increased parasite load of *Leishmania amazonensis* in BALB/c but not C57BL/6 macrophages. Microbes Infect.

[19] Chaves MM, Marques-da-Silva C, Monteiro APT, Canetti C, Coutinho-Silva R (2014). Leukotriene B4 modulates P2X7 receptor-mediated *Leishmania amazonensis* elimination in murine macrophages. J Immunol.

[20] Araújo-Santos T, Prates DB, Andrade BB, Nascimento DO, Clarêncio J, Entringer PF, Carneiro AB, Silva-Neto MAC, Miranda JC, Brodskyn CI, Barral A, Bozza PT, Borges VM: **Lutzomyia longipalpis saliva triggers lipid body formation and prostaglandin E**_**2**_**production in murine macrophages.** *PLoS Negl Trop Dis* 2010, **4:**e873. 10.1371/journal.pntd.0000873 PMC2970534 21072234

[21] Soares MB, Titus RG, Shoemaker CB, David JR, Bozza M (1998). The vasoactive peptide maxadilan from sand fly saliva inhibits TNF-alpha and induces IL-6 by mouse macrophages through interaction with the pituitary adenylate cyclase-activating polypeptide (PACAP) receptor. J Immunol.

[22] Prates DB, Santos LD, Miranda JC, Souza APA, Palma MS, Barral-Netto M, Barral A (2008). Changes in amounts of total salivary gland proteins of *Lutzomyia longipalpis* (*Diptera: Psychodidae*) according to age and diet. J Med Entomol.

[23] Gomes NA, Gattass CR, Barreto-De-Souza V, Wilson ME, DosReis GA (2000). TGF-beta mediates CTLA-4 suppression of cellular immunity in murine kalaazar. J Immunol.

[24] Lawrence T, Willoughby DA, Gilroy DW (2002). Anti-inflammatory lipid mediators and insights into the resolution of inflammation. Nat Rev Immunol.

[25] Lefèvre L, Lugo-Villarino G, Meunier E, Valentin A, Olagnier D, Authier H, Duval C, Dardenne C, Bernad J, Lemesre JL, Auwerx J, Neyrolles O, Pipy B, Coste A (2013). The C-type lectin receptors dectin-1, MR, and SIGNR3 contribute both positively and negatively to the macrophage response to *Leishmania infantum*. Immunity.

[26] D’Avila H, Maya-Monteiro CM, Bozza PT (2008). Lipid bodies in innate immune response to bacterial and parasite infections. Int Immunopharmacol.

[27] Melo RCN, D’Avila H, Fabrino DL, Almeida PE, Bozza PT (2003). Macrophage lipid body induction by Chagas disease in vivo: putative intracellular domains for eicosanoid formation during infection. Tissue Cell.

[28] D’Avila H, Freire-de-Lima CG, Roque NR, Teixeira L, Barja-Fidalgo C, Silva AR, Melo RCN, Dosreis GA, Castro-Faria-Neto HC, Bozza PT: **Host cell lipid bodies triggered by*****Trypanosoma cruzi*****infection and enhanced by the uptake of apoptotic cells are associated with prostaglandin E**_**2**_**generation and increased parasite growth.** *J Infect Dis* 2011, **204:**951–961. 10.1093/infdis/jir432 21849292

[29] Freire-de-Lima CG, Nascimento DO, Soares MB, Bozza PT, Castro-Faria-Neto HC, de Mello FG, DosReis GA, Lopes MF (2000). Uptake of apoptotic cells drives the growth of a pathogenic trypanosome in macrophages. Nature.

[30] D’Avila H, Melo RCN, Parreira GG, Werneck-barroso E, Castro-faria-neto HC, Bozza PT, Avila HD, Bozza T (2006). *Mycobacterium bovis bacillus Calmette-Guerin* induces TLR2-mediated formation of lipid bodies: intracellular domains for eicosanoid synthesis in vivo. J Immunol.

[31] Afonso L, Borges VM, Cruz H, Ribeiro-Gomes FL, DosReis GA, Dutra AN, Clarencio J, de Oliveira CI, Barral A, Barral-Netto M, Brodskyn CI (2008). Interactions with apoptotic but not with necrotic neutrophils increase parasite burden in human macrophages infected with *Leishmania amazonensis*. J Leukoc Biol.

[32] Peters-Golden M, Canetti C, Mancuso P, Coffey MJ (2005). Leukotrienes: underappreciated mediators of innate immune responses. J Immunol.

[33] Serezani CH, Aronoff DM, Jancar S, Mancuso P, Peters-Golden M (2005). Leukotrienes enhance the bactericidal activity of alveolar macrophages against *Klebsiella pneumoniae* through the activation of NADPH oxidase. Blood.

[34] Anstead GM, Chandrasekar B, Zhao W, Yang JUE, Perez LE, Melby PC (2001). Malnutrition alters the innate immune response and increases early visceralization following *Leishmania donovani* infection. Infect Immun.

[35] Ibrahim MK, Barnes JL, Anstead GM, Jimenez F, Travi BL, Peniche AG, Osorio EY, Ahuja SS, Melby PC (2013). The malnutrition-related increase in early visceralization of *Leishmania donovani* is associated with a reduced number of lymph node phagocytes and altered conduit system flow. PLoS Negl Trop Dis.

[36] França-Costa J, Van Weyenbergh J, Boaventura VS, Luz NF, Malta-Santos H, Oliveira MC, Santos de Campos DC, Saldanha AC, Dos-Santos WL, Bozza PT, Barral-Netto M, Barral A, Costa JM, Borges VM: **Arginase I, Polyamine, and Prostaglandin E2 Pathways Suppress the Inflammatory Response and Contribute to Diffuse Cutaneous Leishmaniasis.** *J Infect Dis* 2014, doi:10.1093/infdis/jiu455. 10.1093/infdis/jiu455 25124926

